# Association between low handgrip strength and obesity with mortality in peritoneal dialysis patients

**DOI:** 10.1038/s41598-023-28708-8

**Published:** 2023-02-01

**Authors:** Jun Young Do, Seok Hui Kang

**Affiliations:** Division of Nephrology, Department of Internal Medicine, College of Medicine, Yeungnam University, 170 Hyeonchung-Ro, Nam-Gu, Daegu, 42415 Republic of Korea

**Keywords:** Diseases, Nephrology, Risk factors

## Abstract

The association between sarcopenia and obesity in peritoneal dialysis (PD) patients is more complex than that of the general population. The aim of this study was, therefore, to evaluate the association of patient survival with sarcopenia or sarcopenic components and obesity in groups of patients with PD. We retrospectively analyzed a dataset from 199 prevalent PD patients. Measurements including handgrip strength (HGS), appendicular lean mass index, and baseline characteristics, were obtained during the period of study. Patients were divided into four groups according to their HGS and obesity: NH-NO (normal HGS and non-obesity, n = 60), NH-O (normal HGS and obesity, n = 31), LH-NO (low HGS and non-obesity, n = 71), and LH-O (low HGS and obesity, n = 37). The median follow-up interval was 17 months. The Kaplan–Meier curve analysis showed that the LH-O group had the poorest patient survival outcome among the four groups (*P* < 0.001). The NH-NO group had a better patient survival outcome compared with the LH-NO group. Univariate and multivariate Cox regression analyses showed that the LH-O group had the highest mortality rate compared with the other groups. The NH-NO group had lower mortality compared with the LH-NO group. The present study demonstrated that obesity with low HGS was associated with the greatest mortality rate in groups defined by HGS and obesity.

## Introduction

Peritoneal dialysis (PD) is one of most important dialysis modalities for patients with kidney failure, who require renal replacement therapy. Advances in care facilities for patients with PD, such as the understanding of peritoneal physiology, catheter connections, and peritonitis care, have led to improved survival outcomes^1^. Insulin resistance and/or chronic inflammation due to an increase in peritoneal dialysate exposure, and uremic conditions, are associated with the development of sarcopenia, obesity, or both^2^. While sarcopenia and obesity may develop in different organs, the two pathologies are often cross-linked. Previous studies have identified sarcopenic obesity as a result of two pathologies, and a positive association between sarcopenic obesity and poor survival outcome has been identified in general and older populations^3,4^. As patients with dialysis are prone to develop sarcopenic obesity, some research has been focused on the clinical impact of sarcopenic obesity^5–7^.

Sarcopenia and/or obesity are associated with an increase in mortality, both independently and synergistically, in the general population. However, the association between sarcopenia and obesity in dialysis patients is more complex than that of the general population. Obesity is a traditional risk factor for cardiovascular disease, but sarcopenia can be prevented by the presence of obesity as a source of energy storage in these patients^8^. Previous studies have evaluated the clinical impact of sarcopenic obesity in dialysis patients, but the number of studies are limited and the results have not been consistent^5–7^. Furthermore, there is little data regarding the association between patient survival as a hard outcome, and sarcopenic obesity in patients with PD. Further studies are required to identify the clinical impact and survival rates of such patients exhibiting sarcopenic obesity. The aim of this study was, therefore, to evaluate the association of patient survival with sarcopenia or sarcopenic components and obesity in groups of patients with PD.

## Methods

### Study population

We retrospectively re-analyzed a dataset from a previous study^9^. Briefly, the data of all patients with PD who attended a tertiary medical center between September 2017 and November 2020 were collected, and written informed consent was obtained from all the patients. In addition, our study enrolled PD patients after ≥ 3 months of treatment without a history of maintenance hemodialysis or kidney transplantation before PD (n = 214). Clinical or laboratory data including body composition, strength, and patient survival were recorded. Among these, 15 patients were excluded due to missing data (n = 9) or an inability to ambulate due to an amputated limb (n = 6). Therefore, 199 patients undergoing PD were finally analyzed. Measurements including handgrip strength (HGS), appendicular lean mass (ALM) index, and baseline characteristics, were obtained during a routine peritoneal membrane equilibration test during the period of study. Patients were divided into four groups according to their handgrip strength (HGS) and obesity: NH-NO (normal HGS and non-obesity), NH-O (normal HGS and obesity), LH-NO (low HGS and non-obesity), and LH-O (low HGS and obesity). The end point of follow-up measurements was December 2021. All mortality events were retrieved from patient medical records. Patients with kidney transplantation, switch to hemodialysis, recovery of renal function, or transfer to other hospitals were defined as censored data at the end of PD. This study received ethical approval from the Yeungnam University Hospital Institutional Review Board and was conducted in accordance with the principles of the World Medical Association Declaration of Helsinki (Approval no: 2020-06-002).

### Baseline variables

We collected baseline data for age, sex, presence of diabetes mellitus (DM), use of automated PD, duration of dialysis (months), body mass index (BMI, kg/m^2^), weekly Kt/V_urea_, C-reactive protein (CRP) level (mg/dL), urine volume (mL/day), serum calcium (mg/dL), phosphorus (mg/dL), sodium (mmol/L), potassium (mmol/L), serum albumin (g/dL), fasting blood glucose (mg/dL), systolic blood pressure (mmHg), diastolic blood pressure (mmHg), triglycerides (mg/dL), high-density lipoprotein levels (mg/dL), normalized protein equivalent for total nitrogen appearance (nPNA, g/kg/day), alkaline phosphatase (IU/L), and intact-parathyroid hormone (pg/mL). All laboratory studies were performed after an overnight fast and dialysate drainage. DM was defined based on a patient-reported history of DM and its diagnosis on medical records, or use of DM medications. The dialysate per serum creatinine level at 4 h (DP4Cr) ratio was obtained during the peritoneal membrane equilibration test and weekly Kt/V_urea_ was calculated using 24-h urine and dialysate collections, as previously described^10^.

### Assessment of body composition, strength, and metabolic syndrome

Body compositions were measured using dual-energy X-ray absorptiometry (DEXA, Hologic, Madison, WI, USA). The measurements were performed after dialysate drainage, with the patients in a supine position, wearing a light gown. Lean mass and fat mass (FM) were measured using the DEXA. The ALM index (kg/m^2^) was defined as the sum of lean mass in the upper and lower extremities divided by height squared. The total FM was defined as whole-body FM. HGS was measured in all the patients using a digital dynamometer (Takei 5401; Takei Scientific Instruments Co., Ltd, Niigata, Japan) and was performed according to an American Society of Hand Therapists protocol^11^. First, the patient maintained an empty abdomen and seated position. Second, the patient positioned the adducted shoulder without rotation, flexed the forearm to 90°, and extended the wrist 0–30°. Each patient performed three trials with the dominant hand. HGS was defined as the highest value among the three trials.

In our study, low HGS was defined as < 28 kg for men and < 18 kg for women from the Asian Working Group for Sarcopenia^12^. Low muscle mass was defined in terms of an ALM index of < 7.0 kg/m^2^ for men and < 5.4 kg/m^2^ for women. In our study, low muscle mass cut-off values were defined based on Asian Working Group for Sarcopenia recommendations^12^. The guideline defines appendicular muscle mass measurements as the appendicular skeletal muscle mass index from the DEXA, but DEXA does not measure the skeletal muscle. Nevertheless, ALM reportedly represents appendicular skeletal muscle, and the ALM index and appendicular skeletal muscle index were used interchangeably in previous studies to derive relevant cut-off values in the guideline^12,13^. Therefore, we defined low muscle mass using ALM index values. Patients with low HGS and ALM index were classified as having sarcopenia. Obesity was defined from previous studies, as the percentage of total FM per body weight > 27% for men and > 38% for women^3,14^.

Metabolic syndrome was defined using a modified version of metabolic syndrome for patients with PD^15^. It was diagnosed when three or more of the following five components were observed: (1) systolic blood pressure ≥ 130 mmHg and/or diastolic blood pressure ≥ 85 mmHg, or drug treatment for hypertension; (2) serum triglycerides ≥ 150 mg/dL, or drug treatment for high triglycerides; (3) high-density lipoprotein level < 40 mg/dL in men or < 50 mg/dL in women, or drug treatment for low high-density lipoprotein; (4) fasting glucose level ≥ 100 mg/dL, or drug treatment for DM; (5) BMI > 25 kg/m^2^.

### Statistical analysis

Data were analyzed using statistical software SAS (version 9.4; SAS, Cary, NC, USA). Categorical variables were expressed in terms of count (percentage) and were analyzed using Pearson’s χ^2^ or Fisher’s exact test. Continuous variables were evaluated for distribution using the Kolmogorov–Smirnov test and were presented as the mean ± standard deviation for data with a normal distribution, and median (interquartile range) for data with a non-normal distribution. Continuous variables with a non-normal distribution were compared using the Kruskal–Wallis test, and those with a normal distribution were compared using a one-way analysis of variance. The Bonferroni or Tukey comparison post-hoc test was used in subgroup comparisons. Survival estimates were calculated using the Kaplan–Meier and Cox regression analyses. In Kaplan–Meier analyses, *P*-values for the comparison of survival curves were determined using the log-rank test. Multivariate Cox regression analyses were adjusted for age and serum albumin based on the statistical significance obtained from the univariate analysis. The proportional hazard assumption was satisfied for all the variables. In addition, we performed competing risk analyses to decrease effects by censored data. For competing risk analyses, we defined censored cases as competing risk and performed the Fine and Gray competing risk model. A *P*-value < 0.05 was considered statistically significant.

## Results

### Participants’ clinical characteristics

The numbers of NH-NO, NH-O, LH-NO, and LH-O were 60 (30.2%), 31 (15.6%), 71 (35.7%), and 37 (18.6%), respectively (Table 1). Groups with low HGS were older than those with normal HGS, and LH-O had the oldest participants among the four groups. Groups with obesity had a greater proportion of males than those without obesity. Groups with low HGS had greater proportions of DM than those with normal HGS. Groups with obesity had greater BMI than those without obesity. Groups with obesity had greater CRP levels than the NH-NO group. Serum albumin levels were greater in groups with normal HGS than those with low HGS. Diastolic blood pressure was lower in the LH-O group than in the NH-NO and NH-O groups. The triglycerides level was highest in the NH-O group. There were no significant differences relating to dialysis modality, dialysis vintages, DP4Cr ratio, urine volume, serum calcium, sodium, potassium, fasting glucose, systolic blood pressure, alkaline phosphatase, and intact parathyroid hormone levels among the four groups. Groups with low HGS had a lower ALM index or HGS than the NH-O group, and it would be associated with male predominance in NH-O group.

**Table 1:** Clinical characteristics of participants.

Characteristics	NH-NO (*n* = 60)	NH-O (*n* = 31)	LH-NO (*n* = 71)	LH-O (*n* = 37)	*P*-value
Age (years)	51 (14)	49 (18)	55 (16)^#^	64 (12)*^#+^	< 0.001
Sex (male)	29 (48.3%)	26 (83.9%)	33 (46.5%)	25 (67.6%)	0.001
Diabetes mellitus (%)	26 (43.3%)	9 (29.0%)	39 (54.9%)	24 (64.9%)	0.015
Dialysis modality (APD, %)	17 (28.3%)	12 (38.7%)	16 (22.5%)	12 (32.4%)	0.377
Dialysis vintage (months)	48 (68)	53 (69)	60 (78)	53 (52)	0.845
Body mass index (kg/m^2^)	23.4 (3.2)	27.2 (4.6)*	23.0 (3.6)^#^	25.2 (5.7)*^+^	< 0.001
Weekly Kt/V_urea_	1.83 (0.36)	1.64 (0.45)	1.94 (0.42) ^#^	1.82 (0.65)	0.020
C-reactive protein (mg/dL)	0.08 (0.25)	0.26 (0.56)*	0.15 (0.33)	0.26 (0.94)*	< 0.001
DP4Cr	0.66 (0.16)	0.59 (0.15)	0.67 (0.17)	0.66 (0.17)	0.393
Urine volume (mL/day)	172 (849)	150 (942)	0 (355)	20 (539)	0.523
Calcium (mg/dL)	8.4 (1.5)	8.6 (0.7)	8.2 (1.1)	8.2 (0.7)	0.084
Phosphorus (mg/dL)	5.5 (1.9)	5.0 (1.3)	4.7 (1.7)	4.4 (1.7)	0.047
Sodium (mEq/L)	137 (4)	139 (7)	136 (4)	137 (5)	0.134
Potassium (mEq/L)	4.7 (0.8)	4.5 (1.1)	4.4 (0.8)	4.8 (1.4)	0.159
Albumin (g/dL)	3.8 (0.5)	4.2 (0.6)	3.5 (0.6)*^#^	3.5 (0.6)^#^	< 0.001
Fasting blood glucose (mg/dL)	116 ± 51	110 ± 36	127 ± 64	124 ± 42	0.373
Sysbolic blood pressure (mmHg)	137 (27)	137 (26)	139 (28)	129 (23)	0.231
Diastolic blood pressure (mmHg)	80 (16)	80 (19)	79 (15)	71 (13)*^#^	0.004
Triglycerides (mg/dL)	146 ± 81	234 ± 133*	138 ± 86^#^	163 ± 102^#^	< 0.001
High-density lipoprotein (mg/dL)	50 (18)	35 (15)	45 (28)*^#^	40 (20)	< 0.001
nPNA (g/kg/day)	0.89 (0.16)	0.76 (0.19)*	0.82 (0.29)	0.75 (0.24)	0.002
Alkaline phosphatase (IU/L)	100 (48)	102 (46)	119 (89)	121 (69)	0.107
i-PTH (pg/mL)	253 (357)	335 (372)	270 (371)	308 (229)	0.522
Percentage of fat mass (%)	25.2 (9.1)	33.2 (5.7)*	26.0 (9.8)^#^	34.1 (9.5)*^+^	< 0.001
ALM index (kg/m^2^)	6.3 ± 1.2	6.8 ± 1.5	5.8 ± 1.0^#^	5.9 ± 1.0^#^	0.002
Handgrip strength (kg)	28.6 (12.9)	32.6 (11.4)	17.4 (8.6)*^#^	18.9 (8.6)*^#^	< 0.001

Data are expressed as mean ± standard deviation for continuous variables with normal distribution and median (interquartile range) for those without normal distribution. Categorical variables are expressed as number (percentage). *P*-values were tested among the four groups and analyzed using Kruskal–Wallis test for continuous variables with non-normal distribution and one-way analysis of variance with normal distribution. Bonferroni correction or Turkey comparison were used for post-hoc comparison between two groups. Categorical data were compared using Pearson’s χ^2^ or Fisher’s exact test.

*NH-NO* patients with normal handgrip strength and without obesity;* NH-O* patients with normal handgrip strength and with obesity;* LH-NO* patients with low handgrip strength and without obesity;* LH-O* patients with low handgrip strength and with obesity; *DP4Cr* four-hour dialysate-to-plasma creatinine concentration ratio; *nPNA* normalized protein equivalent of total nitrogen appearance; *i-PTH* intact parathyroid hormone; *ALM* appendicular lean mass.

*P < 0.05 vs NH-NO

^#^*P* < 0.05 vs NH-O

^+^*P* < 0.05 vs. LH-NO.

### Association between groups and metabolic syndrome

**Figure 1:**
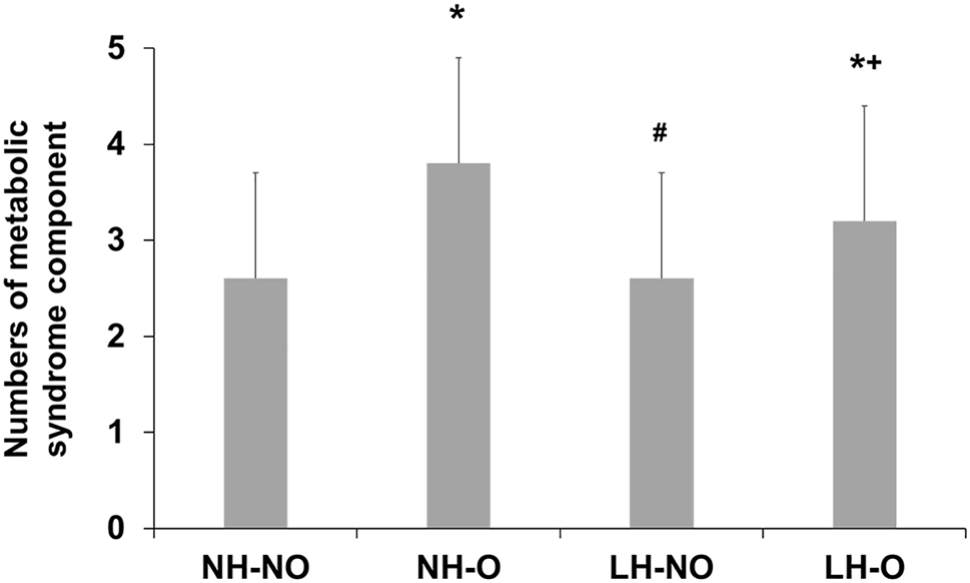
Numbers of metabolic syndrome components according to groups. NH-NO vs NH-O or LH-O, *P* < 0.05; NH-O vs LH-NO, *P* < 0.05; LH-NO vs LH-O, *P* = 0.061; Other comparisons, *P* > 0.100. *P < 0.05 vs NH-NO, ^#^*P* < 0.05 vs NH-O, ^+^*P* < 0.05 vs LH-NO.

The numbers of metabolic syndrome in the NH-NO, NH-O, LH-NO, and LH-O groups were 26 (43.3%), 27 (87.1%), 38 (53.5%), and 29 (78.4%), respectively (*P* < 0.001). The numbers of metabolic syndrome components were 2.6 ± 1.1 in the NH-NO group, 3.8 ± 1.1 in the NH-O group, 2.6 ± 1.1 in the LH-NO group, and 3.2 ± 1.2 in the LH-O group, respectively (Fig. 1, *P* < 0.001). The two groups with obesity had more patients with a metabolic syndrome component relative to the NH-NO group; the NH-O had more patients with a metabolic syndrome component relative to that of the LH-NO group. The 18-month survival rate in patients with or without metabolic syndrome was 87.2% and 89.2%, respectively. The presence of metabolic syndrome was not associated with mortality in our cohort (*P* = 0.314).

### Association between sarcopenia components and survival outcome

The number of patient deaths in the NH-NO, NH-O, LH-NO, and LH-O groups were 1 (1.7%), 1 (3.2%), 12 (16.9%), and 12 (32.4%), respectively (*P* < 0.001). The causes of patient deaths were cardiovascular disease in the NH-NO group and malignancy in the NH-O group. In the LH-NO group, those were cardiovascular disease for 4 patients (33.3%), infection for 6 patients (50%), cachexia for 1 patient (8.3%), and cerebral hemorrhage for 1 patient (8.3%). In LH-O group, those were cardiovascular disease for 7 patients (58.3%), infection for 4 patients (33.3%), and malignancy for 1 patient (8.3%). Total 32 cases were censored. The cause of censoring was kidney transplantation in 12 cases, switch to hemodialysis in 19 cases, and hospital transfer in one case. Median follow-up duration for censored cases were 10 (8) months.

**Figure 2:**
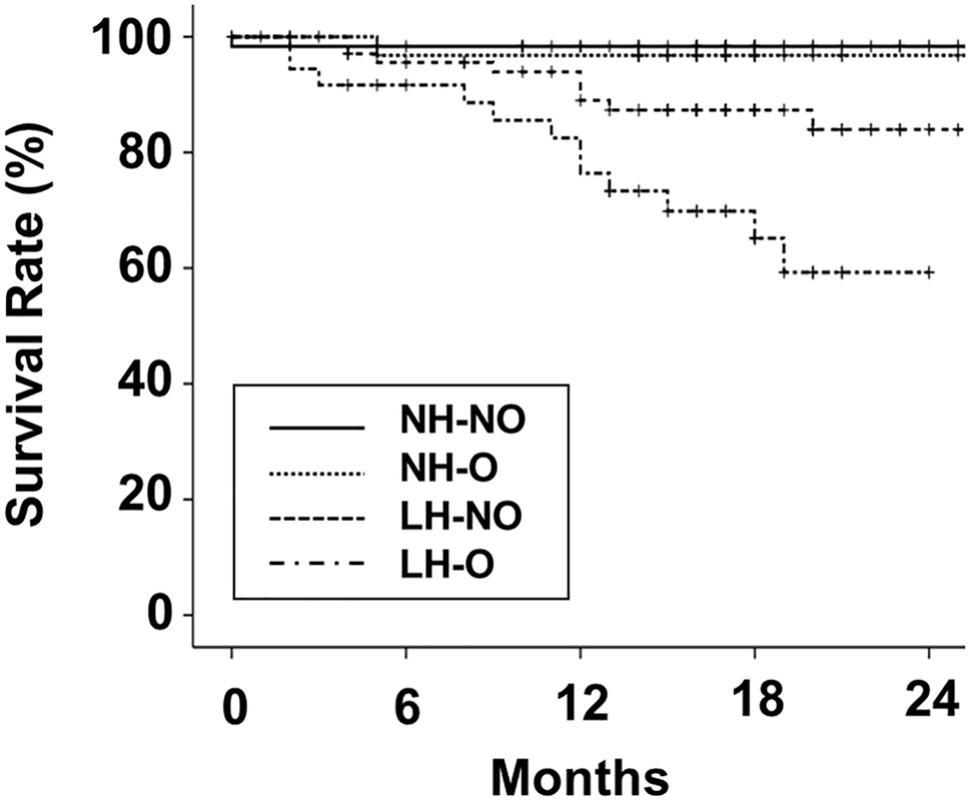
Kaplan–Meier curves of patient survival according to groups. LH-O vs NH-NO, NH-O, or LH-NO, *P* < 0.05; NH-NO vs LH-NO, *P* < 0.05; Other comparisons, *P* > 0.05.

**Figure 3:**
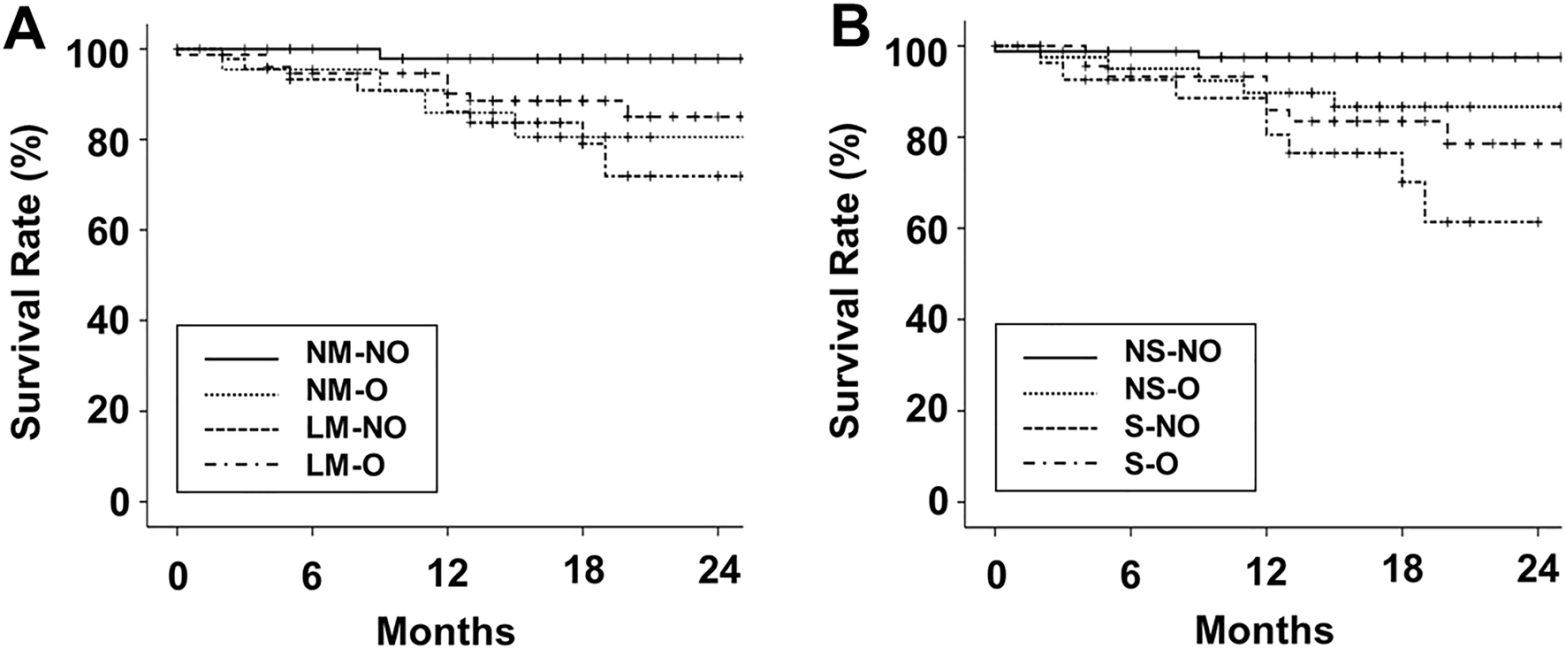
Kaplan–Meier curves of patient survival according to ALM index or sarcopenia. (**A**) The 18-month survival rates in NM-NO, NM-O, LM-NO, and LM-O groups were 97.9%, 80.5%, 88.6%, and 79.1%, respectively (*P* = 0.118). LM-O vs NM-NO or LM-NO, *P* < 0.05; Other comparisons, *P* > 0.100. (**B**) The 18-month survival rates in NS-NO, NS-O, S-NO, and S-O group were 97.5%, 86.6%, 83.5%, and 70.1%, respectively (*P* = 0.003). NS-NO vs S-O or S-NO, *P* < 0.05; Other comparisons, *P* > 0.05.

The follow-up interval was 17 (8) months. The 18-month survival rate in patients with normal or low HGS was 97.8 and 79.7%, respectively (*P* < 0.001). That in patients with or without obesity was 79.8 and 92.8%, respectively (*P* = 0.030). The patient survival was poorer in patients with low HGS or with obesity compared to those with normal HGS or without obesity. These results reveal that combination with HGS and obesity is useful in discriminating the patient survival in patients with low HGS (Fig. S1). The 18-month survival rate was 98.3% in the NH-NO group, 96.8% in the NH-O group, 87.3% in the LH-NO group, and 65.2% in the LH-O group. The Kaplan–Meier curve analysis showed that the LH-O group had the poorest patient survival outcome among the four groups (Fig. 2, *P* < 0.001). The NH-NO group had a better patient survival outcome compared with the LH-NO group. Statistical significances among groups according to ALM index, or sarcopenia and obesity were lower than in those among groups according to HGS alone, and obesity (Fig. 3).

A univariate Cox regression analysis showed that the LH-O group had the highest mortality rate compared with the other groups (Table 2). The NH-NO group had lower mortality compared with the LH-NO group. Multivariate Cox regression analysis showed the same trends as those in the univariate analysis. Subgroup analyses according to age, sex, and the presence of DM showed that the LH-O had the highest mortality rate at the end-point of follow-up in all the subgroups except that of older patients (Table 3). Furthermore, results from the competing risk analysis were similar to those from Cox regression analyses performed using the total cohort (Table S1).

**Table 2:** Cox regression analyses for patient death according to groups.

	Univariate		Multivariate	
	HR (95% CI)	*P*	HR (95% CI)	*P*
Groups by HGS and obesity				
NH-NO (ref: LH-O)	0.03 (0.04–0.25)	0.001	0.05 (0.01–0.40)	0.005
NH-O (ref: LH-O)	0.07 (0.01–0.56)	0.012	0.12 (0.02–0.98)	0.048
LH-NO (ref: LH-O)	0.37 (0.16–0.86)	0.021	0.38 (0.16–0.93)	0.034
NH-NO (ref: NH-O)	0.53 (0.03–8.48)	0.654	0.31 (0.02–6.04)	0.438
NH-NO (ref: LH-NO)	0.09 (0.01–0.66)	0.018	0.12 (0.01–0.93)	0.043
NH-O (ref: LH-NO)	0.20 (0.03–1.51)	0.117	0.28 (0.03–2.34)	0.242
Age (ref: < 65 years)	4.00 (1.84–8.65)	< 0.001	2.10 (0.93–4.72)	0.074
Sex (ref: male)	2.02 (0.91–4.46)	0.082	–	
DM (ref: non-DM)	1.30 (0.60–2.83)	0.504	–	
BMI (per 1 kg/m^2^ increase)	1.02 (0.93–1.12)	0.647	–	
Weekly Kt/Vurea (per increase 1 unit)	0.76 (0.31–1.87)	0.548	–	
CRP (per 1 mg/dL increase)	1.20 (0.98–1.46)	0.073	–	
Phosphorus (per 1 IU/L increase)	0.95 (0.71–1.28)	0.739	–	
Serum albumin (per 1 g/dL increase)	0.37 (0.19–0.74)	0.005	2.14 (0.95–4.86)	0.068
nPNA (per 1 g/kg/day increase)	0.38 (0.05–2.50)	0.316	–	

Multivariate analysis was adjusted for age and serum albumin with *P* < 0.05 in univariate analysis.

*HR* hazard ratio; *CI* confidence interval; *HGS* handgrip strength; *NH-NO* patients with normal handgrip strength and without obesity; *NH-O* patients with normal handgrip strength and with obesity; *LH-NO* patients with low handgrip strength and without obesity; *LH-O* patients with low handgrip strength and with obesity; *DM* diabetes mellitus; *BMI* body mass index; *CRP* C-reactive protein; *nPNA* normalized protein equivalent of total nitrogen appearance.

**Table 3:** The numbers of patient deaths at the end-point of follow-up in the subgroups.

	Sex				Age				DM			
	Men (*n* = 113)		Women (*n* = 86)		< 65 years (*n* = 101)		≥ 65 years (*n* = 98)		Non-DM (*n* = 153)		DM (*n* = 46)	
	No	*P*	No	*P*	No	*P*	No	*P*	No	*P*	No	*P*
NH-NO	0	0.002	1 (3.2%)	0.009	0	0.003	1 (12.5%)	0.313	1 (2.9%)	0.022	0	0.005
NH-O	1 (3.8%)		0		1 (3.6%)		0		1 (4.5%)		0	
LH-NO	2 (6.1%)		10 (26.3%)		7 (13.2%)		5 (27.8%)		6 (18.8%)		6 (15.4%)	
LH-O	7 (28.0%)		5 (41.7%)		5 (25.0%)		7 (41.2%)		4 (30.8%)		8 (33.3%)	

Multivariate analysis was adjusted for age and serum albumin with *P* < 0.05 in univariate analysis.

No., numbers of patient death at the end-point of follow; *P*, *P* -value; *NH-NO* patients with normal handgrip strength and without obesity; *NH-O* patients with normal handgrip strength and with obesity; *LH-NO* patients with low handgrip strength and without obesity; *LH-O* patients with low handgrip strength and with obesity; *DM* diabetes mellitus.

### Association between metabolic syndrome components or obesity and mortality

Among patients with metabolic syndrome, 56 (46.7%) had obesity based on FM and 70 (58.3%) had obesity based on BMI. The proportions of obesity based on two definitions differed. We analyzed patient survival according to high FM (total FM per weight > 27% for men and > 38% for women) based on DEXA measurements and high BMI (> 25 kg/m^2^) based on anthropometric variables. There were 100 (50.3%) patients with a low FM and low BMI, 24 (12.1%) with a high FM and low BMI, 31 (15.6%) with a low FM and high BMI, and 44 (22.1%) with a high FM and high BMI.

Kaplan–Meier curves showed that patients with a low FM and high BMI had better survival than those with a high FM (Fig. S2). Furthermore, we analyzed patient survival according to the presence of metabolic syndrome components. There was no significant intergroup difference in patient survival based on triglycerides, high-density lipoprotein, glucose, or BMI (Fig. S3). For the blood pressure component, patients with high blood pressure had better survival than those with low blood pressure.

## Discussion

Our study included 199 prevalent PD patients and evaluated their body compositions, HGS, and mortality. We divided the patients into various groups based on sarcopenia, sarcopenic components, or obesity. Our study showed that the LH-O group had the poorest patient survival rate among the four groups. Groups defined according to the ALM index, or sarcopenia and obesity were poorer in predicting prognosis as compared to those using HGS and obesity. Univariate and multivariate Cox regression analyses showed that the LH-O group had the highest mortality rate compared to the other groups (Table 2). Results from the subgroup analyses according to age, sex, and the presence of DM were similar to those from the Cox-regression analyses.

Our results revealed that low HGS was associated with patient mortality, regardless of the presence of obesity, but the influence of obesity differed between patients with low and normal HGS. Obesity was not associated with patient survival in patients with normal HGS. Obesity is a well-known risk factor of cardiovascular disease and mortality, but reverse epidemiology is also a well-known phenomenon, in which obesity is paradoxically associated with a favorable outcome in patients with chronic diseases^8^. A time discrepancy between obesity and obesity-related hazard outcomes exist and obesity has a favorable effect on protein energy wasting in patients with chronic diseases on short-term follow-up^16^. Considering the relatively short-term follow-up of our study and the normal HGS in the NH-NO and NH-O groups, one cannot be certain of either the favorable or hazardous effects of obesity. If patients in the NH-NO or NH-O groups do not develop low HGS during further follow-up, one may deduce that obese patients have a poor survival outcome. On the other hand, if patients without obesity develop low HGS later, one may deduce that obese patients have a better survival outcome.

In our study, the association between obesity and metabolic syndrome was evaluated to identify the hazard effect of obesity. We analyzed metabolic syndrome or numbers of its components according to four groups and patients with obesity had greater association with metabolic syndrome compared to those without obesity. There were inconsistent opinions regarding the effect of obesity on patient survival in dialysis patients^16,17^. These inconsistencies have been explained by some factors such as inaccuracies in fat mass measurements and different effects according to dialysis vintage. In our study, obesity was evaluated using DEXA as gold-standard method for predicting fat mass and prevalent PD patients were enrolled. In our study, obesity was inversely associated with patient survival in patients with low HGS. Although our study did not show the association between metabolic syndrome and mortality, the presence of obesity would exert the hazard effect manifested as cardio-metabolic effect in patients with low HGS, which may influence patient survival. Obesity is associated with long-term survival, and it is expected that its cardio-metabolic effects would not be great during short term follow-up. However, obesity can act as an additive effect in patients with sarcopenia, an important comorbidity associated with mortality, and patients with sarcopenia might be made more vulnerable to metabolic complications than those without sarcopenia. This may lead to early hazardous effects of obesity in these patients. Although our study did not evaluate biologic markers or follow-up data, previous studies have shown that the hazardous effects of obesity develop through various underlying mechanisms, such as aggravation of sarcopenia, or chronic inflammation^18^.

There are different definitions for the diagnosis of sarcopenic obesity, and no definite consensus on optimal cut-off values or measurements for predicting muscle mass or obesity has been reached^3–7,15,19,20^. Some studies using different definitions have evaluated the clinical impact or prevalence of sarcopenic obesity in patients undergoing dialysis. Melhotra et al. evaluated 122 patients undergoing hemodialysis and defined sarcopenic obesity using ALM index and percentage of fat mass from DEXA^5^. They did not identify the association between sarcopenic obesity and mortality in these patients. Honda et al. evaluated 328 patients undergoing dialysis and body compositions using DEXA^6^. They showed an inverse association between fat mass and mortality. However, there was no association observed between lean body mass and mortality in their study^7^. Use of ALM index from DEXA could be influenced by volume status. Volume overloading may lead to an overestimation of ALM and an underestimation of sarcopenia. This would attenuate the association between clinical outcome of sarcopenia and sarcopenic obesity, defined using the ALM index of DEXA^21^. Our results also showed a lower association between the low muscle mass group using the ALM index and mortality, compared with those using the HGS. These results are in line with the results from Melhotra’s study^5^. A previous study defined sarcopenic obesity based on low HGS and obesity and showed a positive association between sarcopenic obesity and cardiovascular risk factors in patients with PD^7^. However, the body composition measurements were evaluated using bioimpedance and their study did not have data for survival rates as hard outcomes.

Obesity is an-well known risk factor for mortality in various populations, but metabolic syndrome including obesity as a component was not associated with mortality. In our study, the lack of an association between metabolic syndrome including BMI and mortality would be caused by two issues. First, patients defined as having obesity based on BMI may be associated with favorable outcomes through a high muscle mass. In our study, a high BMI was a component for obesity of metabolic syndrome. However, some patients with a high BMI would have high muscle mass and favorable survival. In contrast, some patients with a low BMI would have a low muscle mass and poor survival. In our study, patients with a low FM and high BMI had better survival than those with a high FM and a high or low BMI, which revealed that the definition of obesity using body composition measurements may be better than that using BMI in high-risk populations of protein energy wasting such as PD patients.

Second, the reverse epidemiology for nutritional status and blood pressure may be associated with the protective effect of some components in metabolic syndrome among PD patients. The reverse epidemiology phenomenon has shown that patients with high cholesterol or blood pressure levels have better survival than those with low cholesterol or blood pressure levels^22^. Our results also showed better survival among patients with a high versus low blood pressure. In addition, there were no significant differences in patient survival according to triglyceride or high-density lipoprotein levels. Although further evaluations of the association between metabolic syndrome and mortality are beyond the scope of this study, the non-association between metabolic syndrome and mortality in PD patients may be associated with mixed effects with both hazardous and protective effects.

Our study has some advantages over previous studies. Patients with PD have been sustainedly prone to volume overload compared to hemodialysis patients with a definite dry weight^23^. Therefore, ALM using DEXA is inherently biased as an overestimation of ALM index and HGS may be more accurate with the volume independent method. Our study also did not show significant differences in the ALM index between the NH-NO and LH-NO or LH-O groups, despite differences in muscle strength, and this may be associated with volume effects. We compared patient survival rates according to different definitions using the ALM index, HGS, or sarcopenia. HGS was the best predictor of mortality. In addition, our study evaluated the body composition measurements of patients with PD using DEXA as a gold-standard method of fat mass estimation^24^. A definition of obesity using DEXA may be superior to definitions of obesity with regard to BMI, bioimpedance, or waist circumference in patients with PD. The BMI does not differentiate between fat mass and muscle mass. The bioimpedance method is not the gold-standard in predicting fat mass in patients with PD. Waist circumference can also be influenced by peritoneal dialysate. Finally, we evaluated patient survival as a hard outcome.

This study has several limitations. The first limitation is our study’s design which was based on a single-center, and retrospective in nature. Second, our study was limited by a small sample size and differences in baseline characteristics. ALM index and HGS in the NH-O group were greater than that in the LH-NO or LH-O groups, and it would be associated with male predominance in the NH-O group. However, we suggest that disproportions in sex and DM would not influence the results of survival analyses owing to classification using a different cut-off value according to sex. We included subgroup analyses to overcome this limitation and the LH-O had the highest mortality rate at the end-point of follow-up in all the subgroups except in that of older patients. In addition, the number of patients in the groups was disproportionate and those in the NH-O and LH-O groups were especially small. These factors limited the full adjustment of covariates and we performed limited multivariate Cox regression analyses without adjustments for all confounding factors. Therefore, to select confounding factor for adjustments of multivariate analyses, we excluded variables without statistical significance in univariate Cox regression analyses. Further studies using more covariates and matched propensity scores are required to overcome this limitation. Third, our study used ALM index and HGS measurements at a single time-point without follow-up data. Finally, our study did not include some important variables such as physical performance.

The present study demonstrated that obesity with a low HGS was associated with the greatest mortality rate in groups defined by HGS and obesity. Therefore, clinicians may evaluate patients in terms of the routine assessment of HGS using the volume-independent method to predict patient survival and by including additional discriminating factors related to obesity in patients with PD.

## Supplementary Information

Supplementary Information.

## Data Availability

All data generated or analyzed during this study are included in this published article and its Supplementary Information files.

## Abbreviations

ALM: Appendicular lean mass
NH-NO: Patients with normal handgrip strength and without obesity
NH-O: Patients with normal handgrip strength and with obesity
NM-NO: Patients with normal ALM index and without obesity
NM-O: Patients with normal ALM index and with obesity
LH-NO: Patients with low handgrip strength and without obesity
LH-O: Patients with low handgrip strength and with obesity
LM-NO: Patients with low ALM index and without obesity
LM-O: Patients with low ALM index and with obesity
NS-NO: Patients without sarcopenia and without obesity
NS-O: Patients without sarcopenia and with obesity
S-NO: Patients with sarcopenia and without obesity
S-O: Patients with sarcopenia and with obesity

## References

[1] Mehrotra R, Devuyst O, Davies SJ, Johnson DW (2016). The current state of peritoneal dialysis. J. Am. Soc. Nephrol..

[2] Sabatino A, Cuppari L, Stenvinkel P, Lindholm B, Avesani CM (2021). Sarcopenia in chronic kidney disease: what have we learned so far?. J. Nephrol..

[3] Baumgartner RN, Wayne SJ, Waters DL, Janssen I, Gallagher D, Morley JE (2004). Sarcopenic obesity predicts instrumental activities of daily living disability in the elderly. Obes. Res..

[4] Janssen I, Heymsfield SB, Ross R (2002). Low relative skeletal muscle mass (sarcopenia) in older persons is associated with functional impairment and physical disability. J. Am. Geriatr. Soc..

[5] Malhotra R (2017). Sarcopenic obesity definitions by body composition and mortality in the hemodialysis patients. J. Ren. Nutr..

[6] Honda H (2007). Obese sarcopenia in patients with end-stage renal disease is associated with inflammation and increased mortality. Am. J. Clin. Nutr..

[7] Tabibi H, As'habi A, Najafi I, Hedayati M (2018). Prevalence of dynapenic obesity and sarcopenic obesity and their associations with cardiovascular disease risk factors in peritoneal dialysis patients. Kidney Res. Clin. Pract..

[8] Park J (2014). Obesity paradox in end-stage kidney disease patients. Prog. Cardiovasc. Dis..

[9] Do JY, Kim AY, Kang SH (2021). Association between phase angle and sarcopenia in patients undergoing peritoneal dialysis. Front. Nutr..

[10] Kang SH, Do JY (2020). Effects of volume status on body composition in incident peritoneal dialysis patients. Eur. J. Clin. Nutr..

[11] Fess EE (1992). Grip strength. Clinical Assessment Recommendation.

[12] Chen LK (2020). Asian Working Group for Sarcopenia: 2019 Consensus Update on Sarcopenia Diagnosis and Treatment. J Am Med Dir Assoc..

[13] Visser M, Fuerst T, Lang T, Salamone L, Harris TB (1999). Validity of fan-beam dual-energy X-ray absorptiometry for measuring fat-free mass and leg muscle mass. Health, Aging, and Body Composition Study–Dual-Energy X-ray Absorptiometry and Body Composition Working Group. J Appl Physiol.

[14] Baumgartner RN (2000). Body composition in healthy aging. Ann. N Y Acad. Sci..

[15] Li PK, Kwan BC, Szeto CC, Ko GT (2008). Metabolic syndrome in peritoneal dialysis patients. NDT Plus.

[16] Park J (2017). Kidney disease and obesity paradox. Kidney Res. Clin. Pract..

[17] Imam TH, Coleman KJ (2019). Obesity and mortality in end-stage renal disease. Is it time to reverse the "reverse epidemiology"-at least in peritoneal dialysis?. J. Ren. Nutr..

[18] Chen Y (2021). Obesity management and chronic kidney disease. Semin. Nephrol..

[19] Baumgartner RN (1998). Epidemiology of sarcopenia among the elderly in New Mexico. Am. J. Epidemiol..

[20] Janssen I, Baumgartner RN, Ross R, Rosenberg IH, Roubenoff R (2004). Skeletal muscle cutpoints associated with elevated physical disability risk in older men and women. Am. J. Epidemiol..

[21] Formica C (1993). Body composition following hemodialysis: studies using dual-energy X-ray absorptiometry and bioelectrical impedance analysis. Osteoporos Int..

[22] Kalantar-Zadeh K, Block G, Humphreys MH, Kopple JD (2003). Reverse epidemiology of cardiovascular risk factors in maintenance dialysis patients. Kidney Int..

[23] van Biesen W (2013). A multicentric, international matched pair analysis of body composition in peritoneal dialysis versus haemodialysis patients. Nephrol Dial Transplant..

[24] Prior BM (1997). In vivo validation of whole body composition estimates from dual-energy X-ray absorptiometry. J. Appl. Physiol..

