# Supplementary material for: Association between low handgrip strength and obesity with mortality in peritoneal dialysis patients
Source: Sci Rep. 2023 Feb 1;13:1852. doi: 10.1038/s41598-023-28708-8 (PMC9892556; doi:10.1038/s41598-023-28708-8)
Supplement: Supplementary file 1 — Supplementary Information. [file 41598_2023_28708_MOESM1_ESM.doc]

**Supplementary Information**

Figure S1. Kaplan–Meier curves of patient survival according to HGS or obesity

Figure S2. Kaplan–Meier curves of patient survival according to FM and BMI

Figure S3. Kaplan–Meier curves of patient survival according to the presence of metabolic syndrome component

Table S1. Cox regression analyses using the competing risk model

**
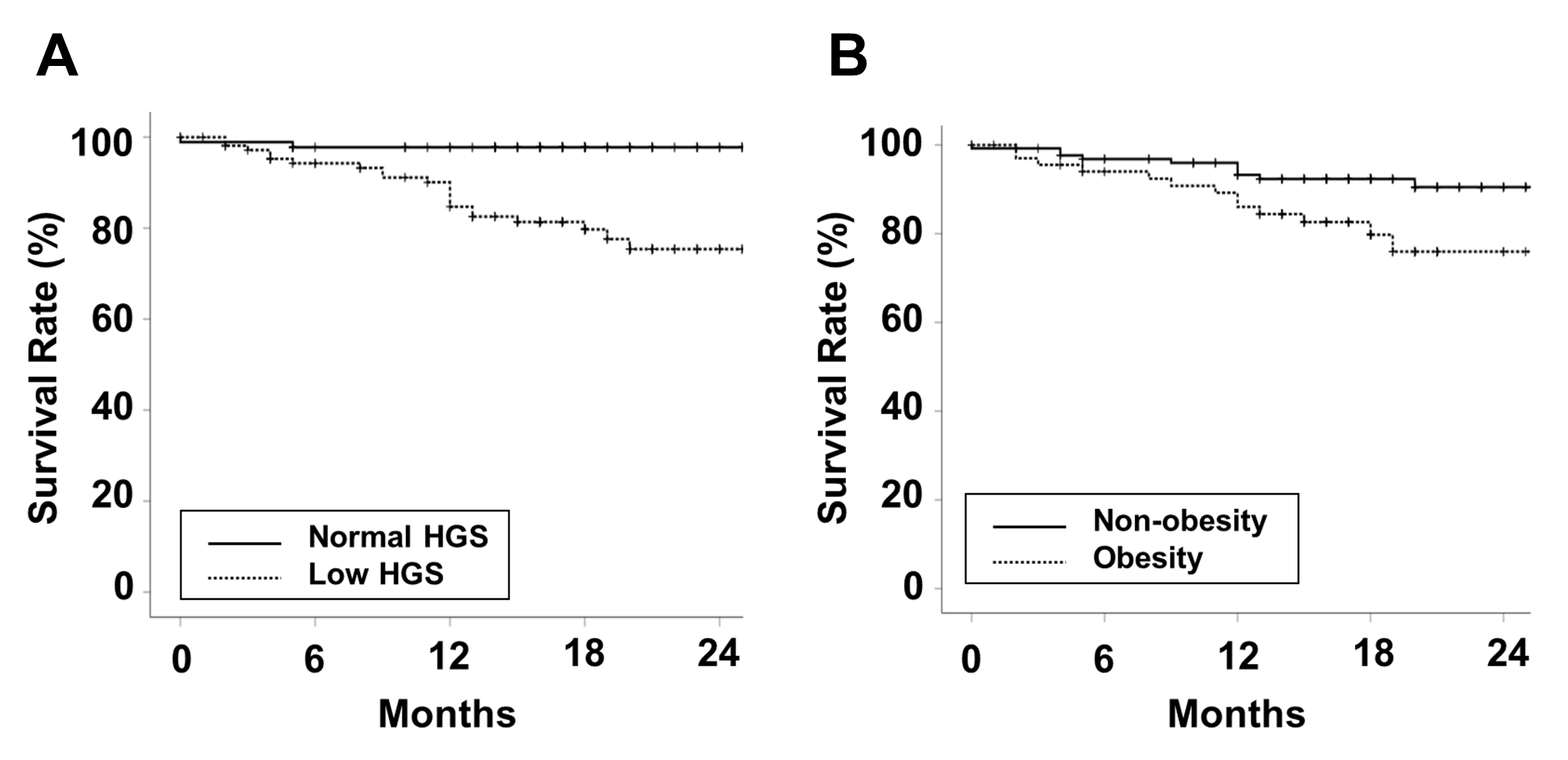
**

**Figure S1. Kaplan**–**Meier curves of patient survival according to HGS or obesity**

**Abbreviations: HGS, handgrip strength**

**
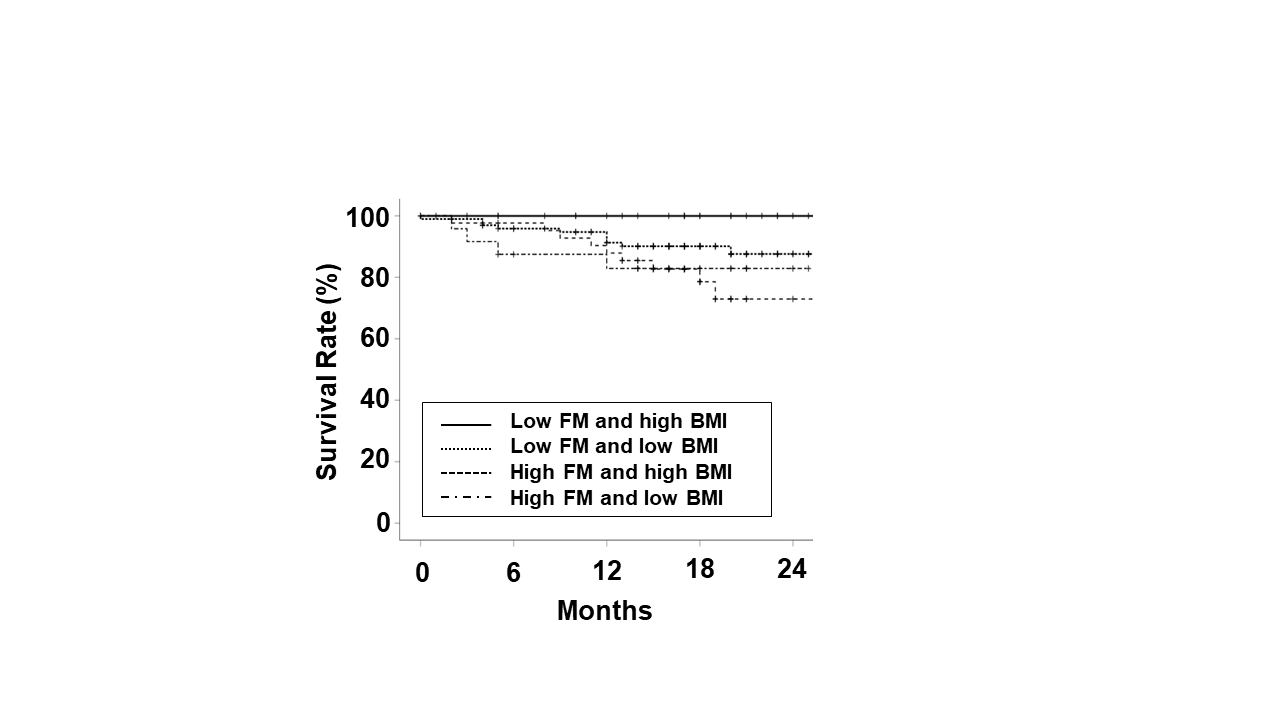
**

**Figure S2. Kaplan–Meier curves of patient survival according to FM and BMI.** The 18-month survival rates of patients with a low FM and high BMI, low FM and low BMI, high FM and high BMI, and high FM and low BMI were 100%, 90.1%, 78.6%, and 82.9%, respectively. Low FM and high BMI vs high FM and low BMI, or high FM and high BMI, *P* < 0.05; Other comparisons, *P* > 0.05.

**Abbreviations:** BMI, body mass index; FM, fat mass

**
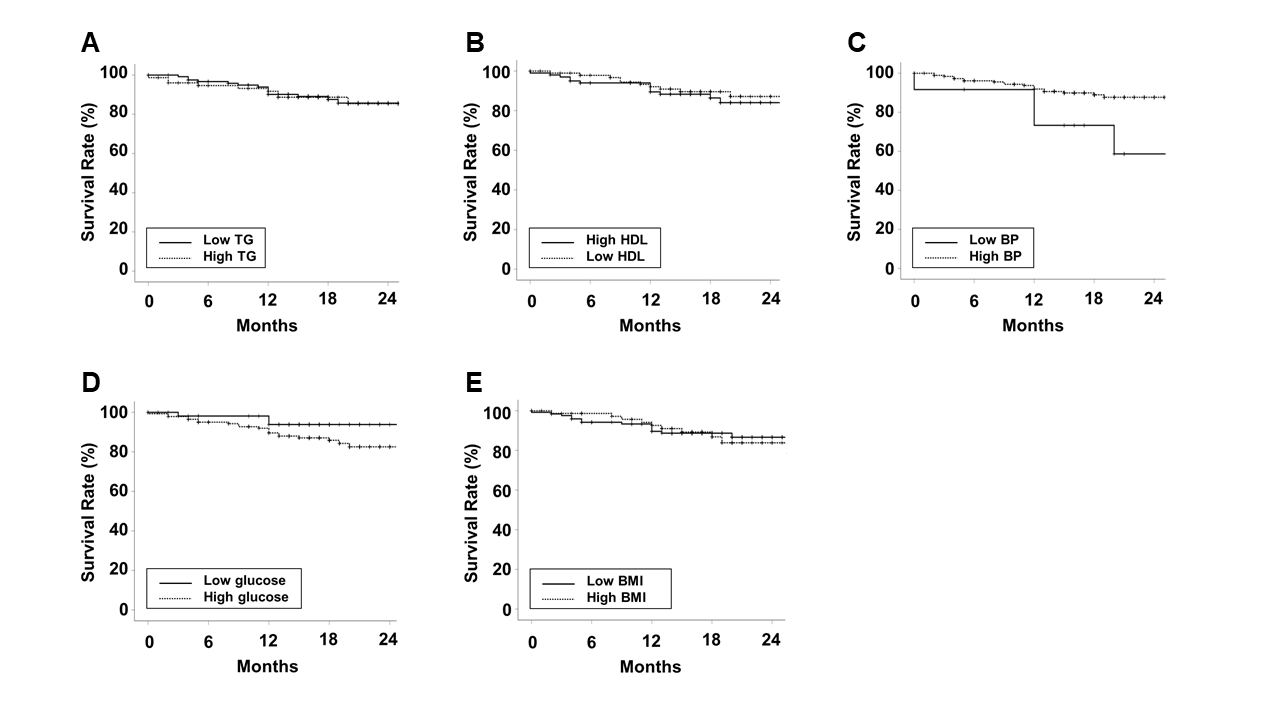
**

**Figure S3. Kaplan–Meier curves of patient survival according to the presence of metabolic syndrome components.** (A) The 18-month survival rates for a low and high TG were 87.6% and 88.7%, respectively (*P* = 0.983). (B) The 18-month survival rates for a high and low HDL were 86.4% and 89.6%, respectively (*P* = 0.983). (C) The 18-month survival rates for a low and high BP were 73.3% and 89.0%, respectively (*P* = 0.036). (D) The 18-month survival rates for a low and high glucose level were 93.8% and 85.9%, respectively (*P* = 0.115). (E) The 18-month survival rates for a low and high BMI were 88.7% and 86.8%, respectively (*P* = 0.659).

**Abbreviations:** BMI, body mass index; BP, blood pressure; HDL, high-density lipoprotein; TG, triglyceride

**Table S1. Cox regression analyses using the competing risk model**

|  | **Univariate** | | **Multivariate** | |
| --- | --- | --- | --- | --- |
|  | **HR (95% CI)** | ***P*** | **HR (95% CI)** | ***P*** |
| Groups by HGS and obesity |  |  |  |  |
| NH-NO (ref: LH-O) | 0.04 (0.04–0.28) | 0.002 | 0.05 (0.01–0.40) | 0.005 |
| NH-O (ref: LH-O) | 0.07 (0.10–0.56) | 0.012 | 0.10 (0.01–0.89) | 0.038 |
| LH-NO (ref: LH-O) | 0.41 (0.17–0.95) | 0.037 | 0.48 (0.20–1.16) | 0.101 |
| NH-NO (ref: NH-O) | 0.66 (0.04–10.54) | 0.768 | 0.54 (0.03–10.48) | 0.681 |
| NH-NO (ref: LH-NO) | 0.09 (0.01–0.67) | 0.019 | 0.11 (0.01–0.93) | 0.042 |
| NH-O (ref: LH-NO) | 0.18 (0.02–1.38) | 0.098 | 0.24 (0.03–2.13) | 0.202 |
| Age (ref: < 65 years) | 3.66 (1.69–7.94) | 0.001 | 1.86 (0.80–4.28) | 0.147 |
| Sex (ref: male) | 1.99 (0.90–4.38) | 0.090 | – |  |
| DM (ref: non-DM) | 1.34 (0.62–2.90) | 0.461 | – |  |
| BMI (per 1 kg/m^2^ increase) | 1.01 (0.93–1.11) | 0.762 | – |  |
| Weekly Kt/Vurea (per increase 1 unit) | 0.76 (0.31–1.88) | 0.557 | – |  |
| CRP (per 1 mg/dL increase) | 1.37 (1.08–1.72) | 0.008 | 1.55 (1.10–2.18) | 0.013 |
| Phosphorus (per 1 IU/L increase) | 0.94 (0.69–1.27) | 0.681 | – |  |
| Serum albumin (per 1 g/dL increase) | 0.25 (0.11–0.58) | 0.001 | 0.56 (0.22–1.41) | 0.220 |
| nPNA (per 1 g/kg/day increase) | 0.41 (0.06–2.69) | 0.351 | – |  |

Multivariate analysis was adjusted for age, CRP, and serum albumin with *P* < 0.05 in univariate analysis.

**Abbreviations**: HR, hazard ratio; CI, confidence interval; HGS, handgrip strength; NH-NO, patients with normal handgrip strength and without obesity; NH-O, patients with normal handgrip strength and with obesity; LH-NO, patients with low handgrip strength and without obesity; LH-O, patients with low handgrip strength and with obesity; DM, diabetes mellitus; BMI, body mass index; CRP, C-reactive protein; nPNA, normalized protein equivalent of total nitrogen appearance
